# Mammalian Non-CpG Methylation: Stem Cells and Beyond

**DOI:** 10.3390/biology3040739

**Published:** 2014-11-11

**Authors:** Sara E. Pinney

**Affiliations:** 1 Department of Pediatrics, Perelman School of Medicine at the University of Pennsylvania, Philadelphia, PA 19104, USA; E-Mail: pinneys@email.chop.edu; Tel.: +1-215-590-3174; Fax: +1-215-590-3053; 2 Division of Endocrinology and Diabetes, The Children’s Hospital of Philadelphia, 3515 Civic Center Boulevard, Philadelphia, PA 19104, USA

**Keywords:** Keywords: non-CpG methylation, epigenetics, bisulfite sequencing, MeDIP, RRBS1

## Abstract

Although CpG dinucleotides remain the primary site for DNA methylation in mammals, there is emerging evidence that DNA methylation at non-CpG sites (CpA, CpT and CpC) is not only present in mammalian cells, but may play a unique role in the regulation of gene expression. For some time it has been known that non-CpG methylation is abundant in plants and present in mammalian embryonic stem cells, but non-CpG methylation was thought to be lost upon cell differentiation. However, recent publications have described a role for non-CpG methylation in adult mammalian somatic cells including the adult mammalian brain, skeletal muscle, and hematopoietic cells and new interest in this field has been stimulated by the availability of high throughput sequencing techniques that can accurately measure this epigenetic modification. Genome wide assays indicate that non-CpG methylation is negligible in human fetal brain, but abundant in human adult brain tissue. Genome wide measurement of non-CpG methylation coupled with RNA-Sequencing indicates that in the human adult brain non-CpG methylation levels are inversely proportional to the abundance of mRNA transcript at the associated gene. Additionally specific examples where alterations in non-CpG methylation lead to changes in gene expression have been described; in *PGC1*α in human skeletal muscle, *IFN-γ* in human T-cells and *SYT11* in human brain, all of which contribute to the development of human disease.

## 1. Introduction

DNA methylation is the biochemical process by which a methyl group is added to the cytosine nucleotide creating a 5-methylcytosine. In mammals, most 5-methylcytosines are found upstream to a guanine nucleotide in DNA and thus this type of DNA methylation is referred to as CpG methylation (C= cytosine, p = phosphate and G = guanine). CpG methylation in mammals is a specific epigenetic mechanism that can contribute to the regulation of gene expression. CpG methylation, along with histone modifications and other modifications that alter nucleosome positioning have been found to affect chromatin availability to transcription factors and other regulatory protein binding, which ultimately can affect the expression of genes. Segments of DNA containing high levels of CpG density, known as CpG islands, have been identified in the promoter regions of the vast majority of human genes. Additional clusters of CpG methylation have been identified in gene bodies, and intergenic regions and it has been hypothesized that these regions have functional roles in regulating gene enhancers and alternative promoters and may contribute to the expression of alternative gene transcripts [1].

In addition to CpG methylation, a methyl group can be added to a cytosine that is not upstream to a guanine and this form of DNA methylation is called non-CpG methylation. In non-CpG methylation, a methyl group is added to a cytosine that is upstream to an adenine, thymine or another cytosine and thus these modifications are referred to as CpA, CpT or CpC methylation. Initial reports of non-CpG methylation were described in *Arabidopsis,* followed by descriptions of non-CpG methylation in eukaryotic DNA [2,3,4]. More recent publications have described significant levels of non-CpG methylation in human embryonic stem cells and in differentiated mammalian cell types including human skeletal muscle and brain [5,6,7,8]. The aim of this review is to summarize what is currently known about non-CpG methylation including current methods used to measure non-CpG methylation and to consider some of the questions that remain to be answered about its role in mammalian cells and development.

## 2. Methods for Detecting and Measuring Non-CpG Methylation

The interest in studies investigating non-CpG methylation has risen greatly over the past few years as the methods to distinguish and accurately measure this DNA modification have improved, especially with the increased availability of high throughput sequencing technology. Initial studies aiming to investigate non-CpG methylation relied on the nearest neighbor assays with nick labeling. This technique provides limited sequence information in the form of dinucleotide composition [3]. In the dual label nearest neighbor analysis protocol, genomic DNA is labeled by 2 different isotopes by “fill in” after restriction enzyme digestion [7,9]. One of the isotopes used for labeling is [α-^32^P] dNTPs incorporating adenine, thymidine and guanine for bases to label for non-CpG methylation (CpA, CpT, CpC) sites [7]. The second isotope used for labeling is [α-^33^P] for CpG methylation [7]. The labels are filled in after the genomic DNA has been digested with methylation sensitive and insensitive restriction enzymes [7,9]. The DNA is digested completely to 3’-dNTPs using micrococcal endonuclease and calf spleen phosphodiesterase exonucleases in order to transfer the [-^32^P] or [-^33^P] from the 5’ position of the labeled nucleotide to the 3’ position of its neighbor [7,9]. The resulting 3’-^32/33^P dNTPs are fractionated by HPLC and quantified [7]. This dual labeling procedure enables the measurement of the frequency of non-CpG methylation directly against the frequency of CpG methylation. Limitations with the method include limited specificity due to measurement of radioactive isotopes including background radioactivity and the large amount of starting material required (5 ug of genomic DNA) for this assay. In addition, this method cannot distinguish CpG or non-CpG methylation results in RNA or contaminating DNA from other species. However, this method does not require sequencing of the product to detect non-CpG methylation and therefore was a historically important technique used in early experiments for studying this subject.

Other methods for detecting non-CpG methylation include methylation sensitive restriction endonuclease (MSRE)-based methods, bisulfite sequencing, methylated DNA immunoprecipitation, and methyl binding domain capture, all of which require PCR amplification or sequencing of the product either at a specific genetic locus of interest or via high throughput methods for genome wide analysis. Treating genomic DNA with bisulfite deaminates unmethylated cytosines under acidic conditions and then leads to a chemical conversion to uracil at alkaline pH. 5-methylcytosine is not sensitive to this treatment. The bisulfite conversion creates non-complimentary DNA strands that are amplified via PCR requiring separate primers for each strand. These primers must be carefully designed as there is an amplification bias for DNA that is unmethylated at non-CpG sites [10]. Incomplete bisulfite conversion of the unmethylated cytosine will give false positive methylation detection. However, incomplete conversion of unmethylated cytosines can be accounted for by incorporating a measured fraction of unmethylated DNA (*i.e.*, lambda DNA) with the experimental sample and calculating the rate of conversion of this known standard [11]. High throughput sequencing of bisulfite treated DNA is computationally intensive since it requires comparison between bisulfite treated and untreated DNA at each base to determine both the CpG and the non-CpG methylated sites, but once the bioinformatic pipelines are established, this method will reveal detailed information about both genome wide CpG and non-CpG methylation patterns at single base resolution. Hairpin-bisulfite PCR allows the detection of cytosine methylation patterns on complementary strands of DNA using a hairpin linker that is targeted and ligated to restriction enzyme-cleaved genomic DNA, thus allowing it to maintain attachment of the complementary DNA strands during the bisulfite conversion and PCR amplification [12] or sequencing [13].

MSRE-based assays use restriction enzymes that digest DNA based on specific recognition sites. Certain restriction enzymes will digest at methylated cytosines and others are insensitive to digestion at cytosines with methylation. Recent publications identified several methylation sensitive restriction enzymes that digest only at non-methylated non-CpG sites including Psp61 or Ajnl and these specific enzymes can be used to quantify non-CpG methylation [6,14]. Combining MSRE based DNA digestion with quantitative PCR using primers that flank the recognition sites of the specific restriction enzyme or high throughput sequencing allows for comparison of PCR products before and after enzyme digestion. Again, the specificity of MSRE based assays depends on full digestion of genomic DNA with restriction enzymes and incomplete digestion will alter results. The LUMA assay (luminometric-based assay for global DNA methylation) is a restriction enzyme based assay that was recently adapted to detect non-CpG methylation [15]. The LUMA assay uses methylation sensitive and insensitive restriction enzymes to digest genomic DNA, followed by quantifying the resulting number of cuts from the restriction enzymes using a luminometric polymerase extension platform (*i.e.*, pyrosequencer) which serves as the experimental readout [6,15]. However, the LUMA method is unable to identify specific positions in the genome where methylation is located and thus represents a global methylation analysis. The LUMA assay can be tailored to detect non-CPG methylation based on the restriction enzymes used [10].

Reduced representation bisulphite sequencing (RRBS) is a method to measure genome wide DNA methylation that incorporates both restriction enzymes and bisulfite sequencing and is enriched for CpG rich areas in order to control the high cost of sequencing the entire genome. This assay has been enriched for CpG dense promoter regions and repeated elements but the main advantage is that the assay output limits the amount of DNA requiring high throughput sequencing. Ziller *et al.* found that RRBS provides no detectable bias toward CpA and CpT dinucleotides and a 2-fold enrichment for CpC dinucleotides [16]. When comparative analysis was performed using both whole genome bisulfite sequencing and RRBS for the H1 p25 human embryonic stem cell (hESC) line, Ziller *et al.* reported that whole genome bisulfite sequencing detected 250,000 non-CpG loci while RRBS detected 213,000 non-CpG loci with and overlap of only 52,000 loci [16]. While RRBS detects a significant number of non-CpG loci in its analysis, the overlap between RRBS and whole genome sequencing is much greater for CpG loci as per its design [16]. For H1 p25 hESCs, whole genome sequencing detected 830,000 CpG sites while RRBS detected 807,000 sites with an overlap of 801,000 CpG loci [16]. While RRBS allows for a limited analysis of non-CpG methylation, its bias towards detection of CpG loci suggests that other methods may be more sensitive for measuring non-CpG methylation.

There are two commonly used approaches to enrich for methylated DNA regions of the genome: methylated DNA immunoprecipitation (MeDIP) and methyl-CpG binding domain (MBD) protein capture. Both techniques can be used to enrich for methylated DNA sequences and can undergo further analysis with sequencing to detect both CpG and non-CpG methylation [17]. MeDIP is based on immunoprecipitation of single stranded DNA containing one or more CpG sites using a monoclonal antibody specific to 5MeC^14^. The MBD approach captures double stranded methylated DNA fragments and is able to detect different DNA methylation densities based on the salt concentrations used during the elution [18]. In both techniques, the anti-methylated antibodies and MDBs used should be specific for 5-methylcytosine and currently there is little data available about the specificity of approaches for measuring non-CpG methylation. There is some evidence that antibody binding can be affected by the density of methylated cytosines in a particular region of DNA [17]. Reports indicate that MeDIP is effective for enriching methylated areas with low CpG density, while MBD capture favors areas with high CpG density and identifies the greatest proportions of CpG islands [18,19]. The majority of publications using methylated DNA immunoprecipitation have been used to describe generalized cytosine methylation. If the binding affinity of the methylated antibody is the same between the CpG methylated sites and the non-CpG methylated sites, the sequencing data could help to determine which immunoprecipitated loci represent CpG methylation and which represent non-CpG methylated loci. However, if both CpG and non-CpG sites are contained within the vicinity of antibody binding, it may be impossible to determine if the methylated antibody was binding at a CpG or a non-CpG site. If an antibody specific to non-CpG methylation were developed, this method would require less high throughout sequencing and decreased bioinformatic analysis compared to current bisulfite sequencing assays. However, there are no antibodies specific to non-CpG methylation available at this time.

## 3. DNA Methyltransferases and Their Roles in Non-CpG Methylation

DNA Methyltransferases (DNMTs) are a highly conserved family of proteins that are responsible for the deposition and maintenance of DNA methylation in mammalian development [20]. DNMT1 is the most abundant DNMT in adult cells and it is responsible for methylating the cytosine on the newly synthesized DNA strand while binding to the methylated cytosine on the parent DNA strand. DNMT1 is primarily involved in maintaining established patterns of DNA methylation through mitosis [19]. DNMT3A and DNMT3B in humans (dnmt3a and dnmt3b in mice) are primarily responsible for *de novo* DNA methylation as they do not require hemi-methylated DNA to bind. DNMT3A and DNMT3B show equal affinity to hemi-methylated and non-methylated DNA [21]. DNMT3A is required for the genome wide *de novo* methylation that occurs soon after embryo implantation during mammalian development and loss of either DNMT3A or DNMT3B is lethal to the embryo. DNMT3L is another member of the DNMT3 subgroup and when bound to DNMT3A or DNMT3B the catalytic activity of the complex increases greatly [22,23]. In male germ cells, DNMT3A activity is high during the timing of epigenetic reprogramming and mitotic arrest [11]. DNMT3A, DNMT3B and DNMT3L are considered important in establishing patterns of non-CpG methylation in ESCs [7,13,24]. DNTM3L has increased expression in ESCs compared to somatic cells and its expression seems to parallel the prevalence of non-CpG methylation in the specific tissue type [24].

Experiments using purified recombinant dnmt3a and dnmt3b demonstrate that these enzymes exhibit non-CpG methylation activity *in vitro* [25]. After accounting for background methylation activity, the *in vitro* experiments with recombinant dnmt3a show that CpA and CpT were methylated to 7% and 1% of the CpG methylation level. Comparison studies showed that *in vitro* recombinant dnmt3b1 methylated CpA and CpT to 28% and 46% of the CpG methylation level, indicating that dnmt3b is able to induce more non-CpG methylation that dnmt3a *in vitro* [25].

## 4. Non-CpG Methylation in Mammalian Gametes, Embryonic Stem Cells and Pluripotent Cells

In 2001, Haines *et al.* examined allele specific non-CpG methylation at the murine *NF1* locus [26]. It is generally believed that global remethylation of the embryonic genome occurs after implantation of the embryo, followed by gene specific demethylation events associated with transcriptional activation according to a specific developmental program [27,28]. In this context, known parent of origin effects have been observed at the human *NF1* gene locus, which further led to the examination of methylation patterns of the highly homologous murine *NF1* gene in both gametes and the earliest stages of preimplantation development. Point mutations or small deletion or insertions were previously shown to be primarily of paternal origin while larger deletions, although less common, have been shown to be primarily of maternal origin [29,30]. The authors found that the murine equivalent of the human exon 31 mutation site was fully methylated in sperm, oocytes and the fertilized embryo up to the 2-cell stage with no allele specific bias detected. However, they found that there was an unexpected allele specific bias in non-CpG methylation at the exon 31 region of the *NF1* locus. Using bisulfite sequencing directed to the region of interest at exon 31 of the *NF1* locus, non-CpG methylation was detected in oocytes but not in sperm. Further analysis showed that non-CpG methylation was present in about 50% of the clones in the 2-cell embryo suggesting a parent of origin effect. Therefore, a strategy for measuring allele specific non-CpG methylation was employed, and the authors were able to show that high levels of non-CpG methylation came from the maternal allele in the 2-cell embryo while very little was attributable to the paternal allele. In addition, comparison between the non-CpG methylation sites in the oocyte and the maternal allele of the 2-cell embryo indicated that additional CpA sites were methylated in the 2-cell embryo, thus implicating that *de novo* non-CpG methylation had occurred. Non-CpG methylation at the *NF1* locus did not persist after the 2-cell embryo stage.

Ichiyanagi *et al.* showed that non-CpG methylation was also present in mouse male germ cells within and around the B1 retrotransposon sequences interspersed within the mouse genome [11]. The non-CpG methylation accumulates in the mitotically arrested prospermatogonia and reaches the highest level by birth in a dnmt3a dependent manner. The degree of non-CpG methylation decreases once mitosis resumes while CpG methylation remains prevalent. The cells eventually loose all non-CpG methylation by the time they become spermatogonia [11]. Kobayashi *et al.* performed genome wide DNA methylation analysis with shotgun bisulfite sequencing in mouse male and female primordial germ cells and their derivative cells at embryonic days 10.5, 13.5 and 16.5 and found non-CpG methylation occurred only in the cells that developed into male gonocytes [31].

Shirane *et al.* found that non-CpG methylation was present in mouse germinal vesicle oocytes (GVOs) as measured by amplification-free whole genome bisulfite sequencing [32]. The authors mapped genome wide methylation for GVOs, and non-growing oocytes (NGOs) [32] and found that the distribution of non-CpG methylation closely resembled the pattern of CpG methylation throughout the genome and was highly enriched at gene bodies [32]. GVOs had up to four times the amount of non-CpG methylation compared to NGOs, indicating that non-CpG methylation is more prevalent in the setting of active oocyte growth [32]. In mutant GVOs lacking dnmt3a and dnmt3l, there was a global reduction in both CpG and non-CpG methylation indicating that the *de novo* DNA methyltransferases, dnmt3a and dnmt3l are both necessary for non-CpG methylation in the growing oocyte [32]. These reports indicate that both developmental stage and parent of origin effects contribute to non-CpG methylation patterns in early development [26].

In 2009, using single base resolution maps of DNA methylation through bisulfite sequencing, Lister *et al.* found that 25% of all cytosine methylation in a hESC line was at non-CpG sites, suggesting that hESCs may use different methylation mechanisms to affect gene regulation [33]. The same authors studied 2 different human differentiated cell lines, and found that non-CpG methylation is lost and 99.98% of the cytosine methylation detected via bisulfite sequencing assay was due to CpG methylation [33]. In the hESCs, non-CpG methylation was enriched in gene bodies and was decreased at the transcription start site, enhancer regions and other protein binding sites [33]. Combining the methylation data with RNA-Seq analysis, the authors found a strong correlation with non-CpG methylation and gene activity within the gene body but no correlation between CpG methylation density and gene expression [33]. The authors concluded that the exclusivity of non-CpG methylation in hESCs that is not observed in differentiated cells suggests that it may have a key role in the origin and maintenance of the pluripotent state. Chen *et al.* compared three different hESC lines and found that heavily methylated non-CpG sites are conserved and the motif *TACAG* is particularly enriched in conserved highly methylated non-CpG sites [34].

Laurant *et al.* in 2010 described a whole genome comparative view of DNA methylation quantified by bisulfite sequencing using three cultured cell types representing three stages of progressive differentiation of human cells: hESCs, a fibroblastic differentiated derivative of hESCs and neonatal fibroblasts as well as published genome wide methylation data from a fully differentiated adult human cell line derived from peripheral mononuclear cells [35]. The authors found that the degree of global DNA methylation was inversely correlated to the differentiation status and that there was a bimodal distribution of methylated CpG dinucleotides. Laurant *et al.* demonstrated that hESCs had the highest level of non-CpG methylation compared to more differentiated cell types and that 20% of all cytosine methylation was attributed to non-CpG methylation in hESCs [35]. The percentage of cytosine methylation attributed to non-CpG methylation gradually decreased as differentiation increased and only <10% was measured as non-CpG methylation in the fully differentiated monocyte cell line [35]. Methylation at CpA dinucleotides was the predominant form of non-CpG methylation in hESC accounting for approximately 10% of the total cytosine methylation at the most undifferentiated state [35]. The percentage of cytosine methylation attributed to CpA methylation decreased as the cell lines became more differentiated and in the fully differentiated monocyte cell line, only about 2% of the total cytosine methylation was attributed to CpA methylation [35]. Other forms of non-CpG methylation were measured in all cell lines studied but the percentage of cytosine methylation attributed to CpT and CpC remained fairly constant even as the cell lines became more differentiated [35]. The CpA methylation density profile measured in these cells lines was similar to the CpG methylation profile; both varieties of cytosine methylation showed hypomethylation in promoter regions of transcribed genes and consistent methylation across the gene body [35].

Another manuscript comparing the role of non-CpG methylation in cell differentiation and maturation was published by White *et al.*, and described the differential patterns of methylation at the interferon-γ (*IFN-γ*) promoter at CpG and non-CpG sites in human neonatal and adult T cells [36]. IFN-γ plays an important role in immunological homeostasis and in particular in the activation of TH1 associated immune functions, which are critical to host defenses against viral and bacterial infections [36]. However, excessive or prolonged production of IFN-γ can also contribute to the pathogenesis of inflammatory diseases as a direct result of the toxic effects of IFN-γ on host tissues or through its role in activating cytotoxic effector cells such as macrophages. At the feto-maternal interface, excessive production of IFN-γ is a major cause of fetal loss [37,38]. Recent studies have indicated that immune function is biased in the fetus toward a protective TH2 response (and thus TH1 antagonistic) and that this response is prompted through a down-regulation of IFN-γ production [36]. It has been reported that stimulated neonatal lymphocytes produce ten-fold less IFN-γ than adult cells [39]. For this study, the authors used targeted bisulfite sequencing for the *IFN-γ* promoter in neonatal cord blood and peripheral blood mononuclear cells from adults. They found that neonatal CD4+/CD45RO- T cells have increased methylation at both CpG and non-CpG sites within and adjacent to the *IFN-γ* promoter [36]. Stimulated IFN-γ production is reduced 5–10 fold in neonatal CD4+ T cells compared to adult CD4+ T cells, while adult and neonatal CD8+ T cells had similar amounts of IFN-γ and no differences in promoter methylation [36]. Experiments designed to test the effect of overexpression of *DNMT3A* in HEK 293 cells, demonstrated the presence of non-CpG methylation at the *IFN-γ* promoter [36]. The authors speculate that IFN-γ plays an important role in the immune system throughout development from the fetal period through adulthood, and therefore the expression of IFN-γ must be finely controlled [36]. These results indicate that both CpG and non-CpG methylation likely contribute to the regulation of IFN-γ expression during development [36].

Ziller *et al.* used 76 genome-scale methylation maps across pluripotent and differentiated human cell types and confirmed that non-CpG methylation occurs predominantly in pluripotent cells types, that it decreases as differentiation progresses and is almost absent in some somatic cell types [16]. The authors noted that non-CpG methylation patterns reappear with iPS cell reprogramming of somatic cells and their results indicate that there is a strong correlation between non-CpG methylation and *DNMT3* expression levels [16]. Knockdown of *DNMT3A* in hESC lines did not result in an appreciable change in global CpG methylation but did result in a 28%–33% decrease in methylated CpAs [16]. Knockdown of *DNMT3B* resulted in an 82% reduction in methylated CpAs with again no change in CpG methylation [16]. These results confirm that the *de novo* methyltransferases DNMT3A and DNMT3B contribute significantly to non-CpG methylation in hESCs [16]. In summary, Ziller *et al.* conclude that non-CpG methylation is a relatively rare and highly variable DNA modification and that CpA methylation is found in the same geographical regions of the genome as CpG methylation [16].

## 5. Non-CpG Methylation in Fully Differentiated Mammalian Cells

Although much of the early work describing the role of epigenetic modification in human disease was initially described in cancer, epigenetic modifications have been associated with metabolic disease including diabetes and obesity [40,41,42]. Barres *et al.* examined skeletal muscle from humans with type 2 diabetes and using the MeDIP assay identified increased cytosine hypermethylation at the *PGC1*α promoter in diabetic subjects [6]. The methylation levels were negatively correlated with *PGC1*α mRNA levels indicating that increased promoter methylation at this loci in part regulated gene expression [6]. Further analysis of the increased levels of cytosine methylation at the *PGC1*α promoter in muscle cells from diabetic humans with bisulfite sequencing revealed that most of the methylated cytosines were within non-CpG dinucleotides [6]. The bisulfite sequencing revealed that increased cytosine methylation was not present at genes adjacent to *PGC1*α (*DHX15 and GBA3*) [6]. In addition, the authors describe inducing an acute increase non-CpG methylation at the *PGC1*α promoter in human muscle cells by exposure to TNF-alpha or free fatty acids but no changes in non-CpG methylation were induced after exposure to insulin or glucose [6]. The increased non-CpG methylation at the *PGC1*α promoter after exposure to the free fatty acid palmitate, was prevented by selective silencing of *DNMT3B* (but not *DNMT1* or *DNMT3A*) indicating that it is DNMT3B that is required for *de novo* non-CpG methylation [6]. Barres *et al.* present strong evidence that non-CpG methylation is not only present in somatic cells, but that non-CpG methylation plays a role in controlling the expression of *PGC1*α in fully differentiated muscle [6]. Furthermore the levels of non-CpG methylation present can be influenced by environmental stimuli [6].

Epigenetic mechanisms have been hypothesized to contribute to neuronal plasticity, neurogenesis and psychiatric disorders. Varley *et al.* described a large single-base resolution DNA methylation profiling on a diverse collection of 82 human cell lines and tissues using RBBS [43]. They found that non-CpG methylation is particularly prevalent in differentiated adult human brain tissue and is reproducible across many individuals [43]. In addition, non-CpG methylation in human adult brain occurred at a different set of loci than the loci observed in human embryonic stem cells [43]. These findings were supported by Lister *et al.* who used MethylC Seq and RNA Seq on human embryonic stem cells, human and mouse frontal cortex from fetal and adult samples [44]. They found that non-CpG methylation was negligible in the fetal frontal cortex but was abundant in the adult frontal cortex. Furthermore, genome wide measurement of non-CpG methylation coupled with RNA-Seq indicated that human brain non-CpG methylation levels are inversely proportional to the abundance of mRNA transcript measured at the associated gene [44]. These findings suggest the assumption that non-CpG methylation decreases as differentiation progresses may be oversimplified and that there are fully differentiated tissues where it is likely that non-CpG methylation is involved in regulating gene expression.

Mutations in the gene that codes for MeCP2, a protein that has been well characterized as member of a methylated CpG binding domain family, can lead to deficits in neuronal development and neuronal functions and can lead to Rhett syndrome, a severe neurodevelopmental disorder in humans [5]. In order to investigate the role of non-CpG methylation in neurons, Guo *et al.* examined whole genome bisulfite sequencing from the adult mouse dentate gyrus and found that 25% of all methylated cytosines were at non-CpG dinucleotides [5]. In additional experiments designed to examine whether neuronal non-CpG methylation identified in DNA from the adult mouse dentate gyrus was conserved in other mammals, Guo *et al.* performed targeted direct bisulfite sequencing in the orthologous genomic regions using adult human brain samples. The authors observed highly reproducible levels of both CpG and non-CpG methylation in all orthologous regions studied [5]. These results suggest that there is evolutionary conservation of neuronal non-CpG methylation [5].

In order to investigate whether non-CpG methylation can affect gene transcription, the authors developed an *in vitro* system by methylating GFP-expressing plasmids with bacterial DNMTs and co-transfecting with unmethylated RFP expression plasmids into HEK293 cells and cultured mouse hippocampal neurons [5]. Gene expression was approximated through the reporter assays as the ratio of GFP+/RFP+ and was equally decreased in the plasmids representing both CpG and non-CpG loci [5]. These results suggest that methylation at both CpG and non-CpG loci have the capacity to repress transcription in differentiated mammalian cells including neurons [5]. Finally, the authors examined the binding capacity of recombinant MeCP2 to methylated oligonucleotides by electromobility shift assay and found that recombinant MeCP2 bound to both CpG and non-CpG methylated dinucleotides but the binding affinity for methylated non-CpG sites was lower in the absence of CpG methylated sites [5]. They subsequently performed MeCP2 chromatin immunoprecipitation (ChIP) followed by bisulfite sequencing of the immunoprecipitated DNA and found that MeCP2 ChIP selectively enriched for CpG methylated loci, but non-CpG methylated regions were also highly enriched in the MeCP2 bound chromatin, supporting an *in vivo* relationship between MeCP2 and non-CpG methylation in neurons [5]. Similar to other studies examining the role for DNMTs in establishing and maintaining non-CpG methylation, Guo *et al.* found that non-CpG methylation is established *de novo* during neuronal maturation and DNMT3A is required for active maintenance, even in post-mitotic neurons [5].

Inoue *et al.* examined the role of non-CpG methylation in the development of schizophrenia based on findings at the promoter region of *Synaptotagmin XI (SYT11*) [45]. SYT11 proteins are localized to the synaptic vesicle or the cellular membrane and act as calcium receptors [45]. Although the role of *SYT11* in neural function is not well understood, there is evidence that there is a relationship between expression of *SYT11* and the development of schizophrenia [45]. Sequence analysis showed that non-CpG dinucleotides within the promoter region of *SYT11* are partially methylated [45]. Gel mobility shift assays demonstrated that when the non-CpG cytosine residues are methylated, SP family protein binding was reduced which may contribute to alterations in gene expression [45]. Transient transcription assays using artificially methylated promoter sequences indicated that methylation of non-CpG sites in the SP-binding site and adjacent regions decreased *SYT11* transcription [45]. These results indicate that non-CpG methylation decreases gene transcription in this model by reducing transcription factor binding [45].

## 6. Conclusions

There is now increasing evidence that non-CpG methylation exists not only in developing cells types, but also in a subclass of fully differentiated cells. Non-CpG methylation is particularly abundant in human adult brain tissue and is present at different loci than in hESCs. There are multiple studies indicating there is a role for non-CpG methylation in the regulation of the expression of specific genes, such as *PGC1*α, *IFN-γ* and *SYT11,* but the exact mechanisms involved remain to be fully elucidated. In many experiments, non-CpG methylation appears to be influenced by the activity of the *de novo* DNA methyltransferases, DNMT3A and DNMT3B, but the detailed actions of these proteins need further study. The increased availability of high throughput sequencing and improved bioinformatic algorithms for data processing will allow for more detailed study of the incidence of non-CpG methylation throughout development, variation in non-CpG methylation patterns amongst different tissues, and a better understanding of how non-CpG methylation contributes to the regulation of gene expression.

## References

[1] Jones P.A. (2012). Functions of DNA methylation: Islands, start sites, gene bodies and beyond. Nat. Rev. Genet..

[2] Woodcock D.M., Crowther P.J., Diver W.P. (1987). The majority of methylated deoxycytidines in human DNA are not in the CpG dinucleotide. Biochem. Biophys. Res. Commun..

[3] Gruenbaum Y., Stein R., Cedar H., Razin A. (1981). Methylation of CpG sequences in eukaryotic DNA. FEBS Lett..

[4] Tasheva E.S., Roufa D.J. (1994). Densely methylated DNA islands in mammalian chromosomal replication origins. Mol. Cell. Biol..

[5] Guo J.U., Su Y., Shin J.H., Shin J., Li H., Xie B., Zhong C., Hu S., Le T., Fan G. (2014). Distribution, recognition and regulation of non-CpG methylation in the adult mammalian brain. Nat. Neurosci..

[6] Barres R., Osler M.E., Yan J., Rune A., Fritz T., Caidahl K., Krook A., Zierath J.R. (2009). Non-CpG methylation of the PCGC-1alpha promoter through DNMT3B controls mitochondrial density. Cell Metab..

[7] Ramsahoye B.H., Biniszkiewicz D., Lyko F., Clark V., Bird A.P., Jaenisch R. (2000). Non-CpG methylation is prevalent in embryonic stem cells and may be mediated by DNA methyltransferase 3a. Proc. Natl. Acad. Sci. USA.

[8] Yan J., Zierath J.R., Barres R. (2011). Evidence for non-CpG methylation in mammals. Exp. Cell Res..

[9] Rein T., DePamphilis M.L., Zorbas H. (1998). Identifying 5-methylcytosine and related modifications in DNA genomes. Nucleic Acids Res..

[10] Patil V., Ward R.L., Hesson L.B. (2014). The evidence for functional non-CpG methylation in mammalian cells. Epigenetics.

[11] Ichiyanagi T., Ichiyanagi K., Miyake M., Sasaki H. (2013). Accumulation and loss of asymmetric non-CpG methylation during male germ-cell development. Nucleic Acids Res..

[12] Laird C.D., Pleasant N.D., Clark A.D., Sneeden J.L., Hassan K.M., Maney N.C., Vary J.C., Morgan T., Hansen R.S., Stoger R. (2004). Hairpin-bisulfite PCR: Assessing epigenetic methylation patterns on complementary strands of individual DNA molecules. Proc. Natl. Acad. Sci. USA.

[13] Arand J., Spieler D., Karius T., Branco M.R., Meilinger D., Meissner A., Jenuwein T., Xu G., Leonhardt H., Wolf V. (2012). *In vivo* control of CpG and non-CpG DNA methylation by DNA methyltransferases. PLoS Genet..

[14] Lopez Castel A., Nakamori M., Thornton C.A., Pearson C.E. (2011). Identification of restriction endonucleases sensitive to 5-cytosine methylation at non-CpG sites, including expanded (cag)n/(ctg)n repeats. Epigenetics.

[15] Karimi M., Johansson S., Ekstrom T.J. (2006). Using luma: A luminometric-based assay for global DNA-methylation. Epigenetics.

[16] Ziller M.J., Muller F., Liao J., Zhang Y., Gu H., Bock C., Boyle P., Epstein C.B., Bernstein B.E., Lengauer T. (2011). Genomic distribution and inter-sample variation of non-CpG methylation across human cell types. PLoS Genet..

[17] Fouse S.D., Nagarajan R.O., Costello J.F. (2010). Genome-scale DNA methylation analysis. Epigenomics.

[18] Nair S.S., Coolen M.W., Stirzaker C., Song J.Z., Statham A.L., Strbenac D., Robinson M.D., Clark S.J. (2011). Comparison of methyl-DNA immunoprecipitation (MeDip) and methyl-CpG binding domain (MDB) protein capture for genome-wide DNA methylation analysis reveal CpG sequence coverage bias. Epigenetics.

[19] Robertson K.D., Uzvolgyi E., Liang G., Talmadge C., Sumegi J., Gonzales F.A., Jones P.A. (1999). The human DNA methyltransferases (dnmts) 1, 3a and 3b: Coordinate mRNA expression in normal tissues and overexpression in tumors. Nucleic Acids Res..

[20] Smith Z.D., Meissner A. (2013). DNA methylation: Roles in mammalian development. Nat. Rev. Genet..

[21] Okano M., Bell D.W., Haber D.A., Li E. (1999). DNA methyltransferases dnmt3a and dnmt3b are essential for *de novo* methylation and mammalian development. Cell.

[22] Gowher H., Liebert K., Hermann A., Xu G., Jeltsch A. (2005). Mechanism of stimulation of catalytic activity of dnmt3a and dnmt3b DNA-(cytosine-c5)-methyltransferases by dnmt3l. J. Biol. Chem..

[23] Gowher H., Stockdale C.J., Goyal R., Ferreira H., Owen-Hughes T., Jeltsch A. (2005). *De novo* methylation of nucleosomal DNA by the mammalian dnmt1 and dnmt3a DNA methyltransferases. Biochemistry.

[24] Su A.I., Wiltshire T., Batalov S., Lapp H., Ching K.A., Block D., Zhang J., Soden R., Hayakawa M., Kreiman G. (2004). A gene atlas of the mouse and human protein-encoding transcriptomes. Proc. Natl. Acad. Sci. USA.

[25] Aoki A., Suetake I., Miyagawa J., Fujio T., Chijiwa T., Sasaki H., Tajima S. (2001). Enzymatic properties of *de novo*-type mouse DNA (cytosine-5) methyltransferases. Nucleic Acids Res..

[26] Haines T.R., Rodenhiser D.I., Ainsworth P.J. (2001). Allele-specific non-CpG methylation of the Nf1 gene during early mouse development. Dev. Biol..

[27] Wolffe A.P., Matzke M.A. (1999). Epigenetics: Regulation through repression. Science.

[28] Turker M.S. (1999). The establishment and maintenance of DNA methylation patterns in mouse somatic cells. Semin. Cancer Biol..

[29] Lazaro C., Gaona A., Ainsworth P., Tenconi R., Vidaud D., Kruyer H., Ars E., Volpini V., Estivill X. (1996). Sex differences in mutational rate and mutational mechanism in the NF1 gene in neurofibromatosis type 1 patients. Hum. Genet..

[30] Ainsworth P.J., Chakraborty P.K., Weksberg R. (1997). Example of somatic mosaicism in a series of *de novo* neurofibromatosis type 1 cases due to a maternally derived deletion. Hum. Mutat..

[31] Kobayashi H., Sakurai T., Miura F., Imai M., Mochiduki K., Yanagisawa E., Sakashita A., Wakai T., Suzuki Y., Ito T. (2013). High-resolution DNA methylome analysis of primordial germ cells identifies gender-specific reprogramming in mice. Genome Res..

[32] Shirane K., Toh H., Kobayashi H., Miura F., Chiba H., Ito T., Kono T., Sasaki H. (2013). Mouse oocyte methylomes at base resolution reveal genome-wide accumulation of non-CpG methylation and role of DNA methyltransferases. PLoS Genet..

[33] Lister R., Pelizzola M., Dowen R.H., Hawkins R.D., Hon G., Tonti-Filippini J., Nery J.R., Lee L., Ye Z., Ngo Q.M. (2009). Human DNA methylomes at base resolution show widespread epigenomic differences. Nature.

[34] Chen P.Y., Feng S., Joo J.W., Jacobsen S.E., Pellegrini M. (2011). A comparative analysis of DNA methylation across human embryonic stem cell lines. Genome Biol..

[35] Laurent L., Wong E., Li G., Huynh T., Tsirigos A., Ong C.T., Low H.M., Kin Sung K.W., Rigoutsos I., Loring J. (2010). Dynamic changes in the human methylome during differentiation. Genome Res..

[36] White G.P., Watt P.M., Holt B.J., Holt P.G. (2002). Differential patterns of methylation of the IFN-gamma promoter at CpG and non-CpG sites underlie differences in IFN-gamma gene expression between human neonatal and adult CD45RO- T cells. J. Immunol..

[37] Wegmann T.G., Lin H., Guilbert L., Mosmann T.R. (1993). Bidirectional cytokine interactions in the maternal-fetal relationship: Is successful pregnancy a TH2 phenomenon?. Immunol. Today.

[38] Krishnan L., Guilbert L.J., Wegmann T.G., Belosevic M., Mosmann T.R. (1996). T helper 1 response against leishmania major in pregnant C57BL/6 mice increases implantation failure and fetal resorptions. Correlation with increased IFN-gamma and TNF and reduced IL-10 production by placental cells. J. Immunol..

[39] Wilson C.B., Westall J., Johnston L., Lewis D.B., Dower S.K., Alpert A.R. (1986). Decreased production of interferon-gamma by human neonatal cells. Intrinsic and regulatory deficiencies. J. Clin. Investig..

[40] Park J.H., Stoffers D.A., Nicholls R.D., Simmons R.A. (2008). Development of type 2 diabetes following intrauterine growth retardation in rats is associated with progressive epigenetic silencing of Pdx1. J. Clin. Investig..

[41] Pinney S.E., Jaeckle Santos L.J., Han Y., Stoffers D.A., Simmons R.A. (2011). Exendin-4 increases histone acetylase activity and reverses epigenetic modifications that silence Pdx1 in the intrauterine growth retarded rat. Diabetologia.

[42] Yang B.T., Dayeh T.A., Kirkpatrick C.L., Taneera J., Kumar R., Groop L., Wollheim C.B., Nitert M.D., Ling C. (2011). Insulin promoter DNA methylation correlates negatively with insulin gene expression and positively with HbA(1c) levels in human pancreatic islets. Diabetologia.

[43] Varley K.E., Gertz J., Bowling K.M., Parker S.L., Reddy T.E., Pauli-Behn F., Cross M.K., Williams B.A., Stamatoyannopoulos J.A., Crawford G.E. (2013). Dynamic DNA methylation across diverse human cell lines and tissues. Genome Res..

[44] Lister R., Mukamel E.A., Nery J.R., Urich M., Puddifoot C.A., Johnson N.D., Lucero J., Huang Y., Dwork A.J., Schultz M.D. (2013). Global epigenomic reconfiguration during mammalian brain development. Science.

[45] Inoue S., Oishi M. (2005). Effects of methylation of non-CpG sequence in the promoter region on the expression of human synaptotagmin XI (syt11). Gene.

